# Associations between parental support, social media addiction, and depressive symptoms among early adolescents in Guam

**DOI:** 10.1371/journal.pmen.0000275

**Published:** 2025-06-25

**Authors:** Francis Dalisay, Masahiro Yamamoto, Yoshito Kawabata, Pallav Pokhrel, Scott K. Okamoto

**Affiliations:** 1 College of Journalism and Communications, STEM Translational Communication Center, University of Florida, Gainesville, Florida, United States of America; 2 Department of Communication, University at Albany, State University of New York, Albany, New York, United States of America; 3 Social & Behavioral Sciences Division, College of Liberal Arts & Social Sciences, University of Guam, Mangilao, Guam, United States of America; 4 Population Sciences in the Pacific Program, University of Hawai’i Cancer Center, University of Hawai’i, Honolulu, Hawai’i, United States of America; University for Continuing Education Krems: Universitat fur Weiterbildung Krems, AUSTRIA

## Abstract

Limited research has examined the potential associations between parental support, social media addiction, and depression among early adolescents in the U.S.-Affiliated Pacific Islands (USAPI). The purpose of the present study is two-fold. First, we examine the relationships between parental support, social media addiction, and depressive symptoms among adolescents in Guam, a USAPI in the Western Pacific. Second, we explore whether the potential relationships between the three above-mentioned variables for Guam adolescents would differ based on ethnicity. We analyzed data from two waves of a survey of middle school students in Guam. Total sample size for Wave 1 of the survey was n = 538; total sample size for the Wave 2 survey was n = 507. Results of a cross-lagged panel design showed that Wave 1 parental support was negatively associated with Wave 2 social media addiction, however, the association between Wave 1 parental support and Wave 2 depressive symptoms was not statistically significant. Also, although Wave 1 social media addiction was positively associated with Wave 2 depressive symptoms, Wave 1 depressive symptoms did not associate with Wave 2 social media addiction. The results thus indicated the association between social media addiction and depressive symptoms is not bi-directional for Guam youths. Furthermore, the results from a multi-group path analysis showed that the above results remained consistent across ethnic groups. These findings have implications for the development of interventions aimed at mitigating social media addiction and depression among Guam youths and USAPI youths more broadly.

## 1. Introduction

Epidemiological studies provide compelling evidence that adolescent depression is a global public health problem. A systematic review and meta-analysis of 72 studies conducted between 2001–2021 revealed that 34% of youths globally are at risk for developing depression [1]. In the U.S., a study that analyzed data from the National Survey on Drug Use and Health found that the percentage of adolescents who reported experiencing a major depressive episode within the past year increased from about 8% in 2009 to roughly 16% in 2019 [2]. U.S. youths living in remote and rural communities, because they tend to have less access to mental health services, could be at a particularly greater risk for experiencing depression and other forms of psychological distress [3,4].

The growing prevalence of depression experienced by adolescents corresponds with the widespread proliferation of social media. Social media are ubiquitous in the lives of adolescents. In the U.S., for instance, majority of teens report that they use social media “several times a day,” and some report they are on certain platforms “almost constantly” [5]. Given these rates of such use, social media addiction (SMA) among adolescents has also become an alarming issue. SMA refers to the compulsive or problematic use of social media [6,7]. Research indicates that SMA is positively associated with depression [8].

However, research on SMA and its association with depression remains limited along three important key areas. First, there is a gap in studies examining SMA and its relationship with depression among early adolescents who come from remote and rural communities. Such youths include those who live in the isolated, largely rural, resource-deprived, and ethnically diverse U.S.-Affiliated Pacific Islands (USAPI). The USAPI include American Samoa, the Commonwealth of the Northern Mariana Islands, the Federated States of Micronesia, Guam, the Republic of the Marshall Islands, and the Republic of Palau. There are an estimated half-million people living in the USAPI, and Pacific Islanders are considered among the fastest-growing groups in the United States [9].

Second, focusing on USAPI youths specifically, not many studies have analyzed factors that could decrease or be inversely associated with both SMA and depression. Past research conducted among youths representing general populations suggests that parental support (PS) serves as an important protective factor for adolescents’ development [10]. PS refers to parents’ praises, encouragement, and affection toward a child that could lead the child to feel accepted [11]. It is possible that PS may be negatively related with both SMA and depression among USAPI youths. Third, to date, research on the relationship between SMA and depression remains inconclusive. Specifically, theories and empirical evidence suggest three plausible directions of this relationship: SMA predicts depression, depression predicts SMA, or the association between these two variables is bi-directional. Given these three plausibilities, it is thus important to identify which among them is most applicable to youths of given communities and different ethnic backgrounds, such as those living in the USAPI.

To address the three above-noted research gaps, the purpose of the present study is two-fold. First, we analyze how PS is related to both SMA and depressive symptoms (DS) among early adolescents in Guam, a USAPI located in the sub-region of Micronesia in the Western Pacific. Second, we explore whether the associations between PS, SMA, and DS among Guam adolescents would differ based on ethnicity. We analyze data from two waves of a survey of Guam adolescents.

Before proceeding, we would like to acknowledge that while social media presents social, emotional, and health risks, it may also enhance relational closeness, self-expression, exploration of interest, and entertainment [12]. Existing meta-analyses show that social media use is also positively associated with perceived online social support, positive affect, and well-being, particularly when users actively engage with interactive features [13, 14, 15]. Yet the discourse that surrounds social media use tends to focus on excessive or problematic use, and its potential negative associations with mental health and well-being. We, therefore, should also acknowledge that while we do use the term social media addiction throughout the entire manuscript, we note that currently, it is not recognized as a formal mental disorder by the Diagnostic and Statistical Manual of Mental Disorders, Fifth Edition (DSM-5) or the International Classification of Diseases, 11th Revision (ICD-11). The ICD-11, however, does include a category for Internet Gaming Disorder (IGD). Indeed, our conceptualization and operationalization of SMA were largely derived from van den Ejinden et al.’s work in developing the Social Media Disorder Scale [7]. The development of that scale was based on the DSM-5 diagnostic criteria for IGD.

## 2. Literature review

### 2.1. Guam adolescents and depressive symptoms

We focus on Guam as recent research indicates that residents on the island are prone to experiencing high levels of psychological distress [16]. In particular, data from the Guam State Epidemiological Profile [17] indicate the percentage of high school students who reported they felt sad or hopeless almost daily “for 2 weeks in a row, within the past 12 months” (p. 106), which are symptoms of depression, had increased substantially between the years 2015–2019. According to 2019 Youth Risk Behavior Surveillance System (YRBSS) data, about 47% of high school students in Guam reported feeling sad or hopeless in the past 12 months compared to about 37% of them in the United States as a whole, who felt the same [18]. Around 11% of high school students in Guam reported that they did not go to school because they felt it was unsafe, compared to about 9% of them in the United States [18]. These relatively higher rates of depression and feeling unsafe at school among Guam youth should be considered mental health risks that could interfere with their healthy socialization with their parents and peers. In the more relationship-oriented cultural context of Guam, a mixed-methods study found that relational aggression and peer victimization (i.e., interpersonal harm and manipulation) and feelings of insecurity in relationships played a role in the development of depressive symptoms among young adults [19]. Given these previous findings, it is important to investigate factors that could serve to either protect or put Guam adolescents at risk for developing depression.

### 2.2. Parental support, depression and social media addiction

First, it is possible that for adolescents in Guam, greater levels of PS would be negatively associated with DS. There is robust evidence that PS is associated with lower levels of depression and DS among adolescents [10,11,20–23]. The general benefits model suggests that it is through having access to parental support that adolescents are more equipped and prepared to cope with adversity and stress. This, in turn, could serve to protect adolescents from psychological distress such as depression. A meta-analysis of 341 studies conducted by Rueger et al. [20] found support for the general benefits model, suggesting that PS could reduce depression. Research conducted with rural youths in the U.S. indicates that having positive relationships with both parents and peers is associated with a lesser likelihood of reporting depressive symptoms [4]. To our knowledge, there is currently no prior research on the association between PS and DS among Guam adolescents, and this was confirmed by a thorough literature search. However, research conducted in Hawaii by Wills and colleagues suggests that PS has an overall effect in buffering the positive relationship between experiencing negative life events and substance use among Hawaii adolescents [24]. We should note that the study found that this buffering effect for PS was observed only among Filipino and other Asian adolescents, but not for Caucasian or Native Hawaiian or other Pacific Islander (NHOPI) adolescents. Wills and colleagues explained that for NHOPI youths in Hawaii, particularly, a buffering effect may also come from support from a network of extended family, cousins, and siblings. Such youths belong to communities characterized by interconnected extended family networks (e.g., uncles, aunts, cousins). Additionally, a comprehensive review of research on suicide among U.S. Pacific Islanders living in Hawai’i, American Samoa, and the Micronesia region (located in the Western Pacific) found that having low family support serves as a risk factor for suicide attempts by youths [25].

Second, it is possible that for adolescents in Guam, greater levels of PS will be negatively associated with SMA. As noted above, SMA refers to the compulsive or problematic use of social media [7]. SMA is part of the overarching construct of internet addiction. Two recent studies suggest that parental support is negatively associated with adolescents’ SMA [26,27]. Hwang and Toma [27] found that higher levels of perceived PS are associated with a lesser likelihood of problematic internet use. In a similar vein, Ren and Zhu [26] found that adolescents who perceived their parents to be supportive were also less likely to both spend time online and use the Internet for leisure activities. Ren and Zhu also revealed that adolescents who view their parents as being supportive, also report that their parents play an active role in mediating their online behaviors. These include explaining why some online sites are good or bad, or talking about contents that are and are not appropriate to share online. Furthermore, active parental mediation was inversely associated with the amount of time adolescents spent online [26]. In sum, the general benefits model of social support [20] and the research reviewed above provide evidence that PS could serve as a protective factor against SMA.

### 2.3. Social media addiction and depression

As mentioned, extant research conducted among US adults provides evidence that excessive and problematic social media use is positively related to depression [28, 29, 30]. Although they did not examine social media use specifically, Fang et al.’s [31] recent study summarized theories and empirical research that suggest three possibilities for the direction of the relationship between the problematic use of the internet (PIU) and depression.

First, it is plausible that SMA predicts DS. This possibility is at the heart of displacement theory [31]. According to this theory, as individuals spend more time online, this displaces the time they are able to spend offline with others, including their peers and family members. Recently, however, Hall and Liu [32] have held that there is limited evidence that time spent on social media displaces time spent on face-to-face interactions. Instead, the use of social media might displace other types of media activities. Yet there is evidence that dysfunctional and excessive use of the Internet could lead to Internet addiction, which subsequently, could result in psycho-social health problems [33]. Fan, for instance, found that internet addiction positively predicts psychological distress [34]. Lam and Peng have shown that internet addiction could predict depression [35]. One mechanism that could explain why SMA is positively associated with DS among adolescents is found in the tendency toward social comparison. Specifically, when individuals use social networking sites to compare themselves to others whom they perceive to be more superior—a phenomenon referred to as upward social comparison—this puts them at a greater risk for developing depression [36,37]. Research shows that excessive use of social media often results in using social media platforms for social comparison [38]. As Pantic has held, the proclivity to use social media to compare oneself to others “may lead to incorrect conclusions regarding physical appearance, educational level, intelligence, moral integrity, as well as many other characteristics of online friends” [39]. Similarly, Kim et al. [38] have shown that addiction to social networking sites could increase upward social comparison, which subsequently could result in lower self-esteem.

Second, according to the cognitive-behavioral model of PIU, psychopathologies such as depression are predictive of problematic uses of the Internet and its symptoms. Davis’ conceptualization of the cognitive-behavioral model specifically assumes that psychopathologies (e.g., depression) could result in maladaptive cognitions such as self-doubt and holding a negative view of oneself [40], which could then lead individuals to turn to the Internet to cope. Individuals may come to believe that the Internet, as opposed to offline interaction, provides a non-threatening outlet where they feel accepted and respected. This could then lead to the eventual overuse of the Internet. Based on the cognitive-behavioral model, we can expect that psychopathologies such as depression could result in maladaptive cognitions (e.g., having a feeling of low self-worth), which could then lead individuals to turn to social media to cope. Individuals may perceive social media to be less threatening than offline interactions, which may then lead to the excessive and compulsive use of social media.

Yet recent studies have also found evidence that the association between problematic internet use and depression could also be bidirectional [41,42]. Thus, it is also plausible that changes in depression predict changes in SMA, and vice versa. Adolescents who display depression might be prone to hold maladaptive cognitions (e.g., feeling low self-worth). As a result, they may cope with feelings of depression by using social media, which could then result in the excessive and addictive use of social media. In turn, as adolescents excessively use social media, they may also be led to compare themselves to their online friends. Comparisons to online friends perceived to be more superior, could lead to a sense that oneself lacks in physical appearance, intelligence, and other characteristics [38]. In this case, SMA could then be associated positively with DS. There would then be a continuous cycle between SMA and depression and vice versa.

In sum, the above literature on the protective role of PS suggests that it would be negatively related to both SMA and DS. Also, the literature suggests SMA is positively associated with DS, however, the nature of this relationship could be either unidirectional or bidirectional. Yet given the limited research in this area conducted in Guam, we propose and investigate the following research question for Guam adolescents:

RQ1. How are PS, SMA, and DS related?

### 2.4. Potential role of ethnicity

As noted previously, our study also explores the potential that the associations between PS, SMA, and DS would differ based on ethnicity. According to most recent population statistics [43], CHamorus, who are the native/indigenous people of Guam, and Filipinos, respectively make up around 33% and 29% of the island’s population. As the two largest ethnic groups in Guam, CHamorus and Filipinos in Guam share certain cultural norms and values, which can be attributed to their parallel historical experiences, namely centuries of Spanish and U.S. colonialism [44]. Non-CHamoru Micronesians in Guam, who originate from the Freely Associated States (FAS), are the third largest ethnic group in the island, and make up more than 11% of the population. The FAS group includes indigenous peoples originating from the Federated States of Micronesia (FSM; including Chuukese, Kosraeans, Pohnpeians, and Yapese), the Republic of Palau (Palauans), and the Republic of the Marshall Islands (Marshallese). FAS migrants are allowed to enter Guam or the United States without visa restrictions under the Compacts of Free Association with the United States, which were signed in 1986 for the FSM and the Republic of the Marshall Islands and in 1994 for the Republic of Palau.

While there is scant research relevant to PS and SMA among youths representing these three ethnic groups living in Guam, a study conducted by Bosqui et al. suggested that adult CHamorus and other Pacific Islanders in Guam (namely, those from the FAS) are at a greater risk for experiencing psychological distress than Filipinos [16]. The study’s results also suggested that CHamorus have the greatest risk for psychological distress among the three groups. The researchers partly attributed these findings to the higher rates of poverty and levels of disadvantages experienced by CHamorus and other Pacific Islanders in Guam, compared to Filipinos. In sum, given these potential differences in psychological distress between the three largest ethnic groups in Guam, we investigate the following research question:

RQ2: Will the relationships between PS, SMA, and DS differ based on ethnicity?

## 3. Materials and methods

### 3.1. Ethics statement

The University of Guam Institutional Review Board (IRB) committee, which serves as the regional IRB board, approved this study (approval number for the study is CHRS #22–69). Both written parental consent and student assent were sought before students were allowed to participate in the survey. In addition, permission to conduct the study was granted by the Guam Department of Education (GDOE) and the principals of each of the 8 public middle schools.

### 3.2. Procedure

The present study conducted a secondary analysis of survey data collected for a pilot clinical trial that tested the efficacy of a school-based substance use prevention curriculum for Guam middle school students. We analyzed survey data from Wave 1 (W1; baseline) and Wave 2 (W2; posttest) of the primary study. These surveys were administered from September 30, 2022 through May 9, 2023. We conducted multivariate analyses of variance to test whether there were statistically significant differences in the mean scores between the intervention and control groups along all our key variables in W1 and W2. Because these results were not directly relevant to our main analyses, we do not include them in this study, but we have included them as a supplement (S1 Text).

These preliminary analyses revealed that there were no statistically significant differences (*p* < .05) in the mean scores between the intervention and control groups along all our key variables. However, we included group as a covariate in our models. The average class size for the schools that participated in the study was approximately 15–25 students. Participants were assured that their responses would remain anonymous as the survey did not ask for any identifying information. Surveys were self-administered and facilitated by research assistants. The survey took approximately 30 minutes to complete. As compensation for their time and effort, all students received a $10 gift certificate ($5 for each Wave) to a local store. The survey contained items that measured demographics, substance use, social media addiction, parental support, and depression. This study followed the Strengthening the Reporting of Observational Studies in Epidemiology (STROBE) reporting guideline.

### 3.3. Participants

W1 data were collected during baseline and W2 data were collected one month after baseline. All 8 of Guam’s public middle schools participated in this study. There were a total of n = 538 completed surveys for W1 and n = 507 for W2. The overall response rates were roughly 76% for W1 and about 71% for W2, suggesting that around 5% of the sample dropped out during W2. The participant characteristics for Waves 1 and 2 are reported on S1 Table. Of note, for both Waves, the ages of participants ranged from 10-15 years, with roughly 52% male and 48% female for W1, and roughly 53% male and 47% female for W2 (for comparison, male students comprise of about 52% of the GDOE population as a whole) [45]. The grade levels for both W1 and W2 were roughly similar, although there were slightly less 6th graders in W1 (about 24%) than there were for W2 (about 26%), and slightly more 8th graders in W1 (about 46%) than W2 (about 44%). We should point out, however, that our sample may have over-represented 8th graders, as based on data compiled by the Guam Department of Education, the true population of the island’s public middle schools comprises of roughly 33% 6th graders, 34% 7th graders, and 33% 8th graders [46]. Regarding ethnicity for both W1 and W2 data, roughly 35% were CHamoru, about 31% were Filipino, about 32% were from the Freely Associated States (FAS), and about 2% were other. Comparatively, population-level data from the Guam Department of Education indicate that there are around 44% CHamorus, 22% Filipinos, and about 31% FAS students enrolled in Guam middle schools [46]. Thus, our sample may have over-represented Filipino students while underrepresenting CHamoru students.

### 3.4. Measures

#### 3.4.1. Parental support.

PS was measured by items acquired from Wills and Cleary’s [47] scale assessing the perceived availability of emotional support received from parents. The items asked about the extent to which the following statements are not true to very true (not true = 1, very true = 5): a) I can share my feelings with my parent, b) I feel that I can trust my parent as someone to talk to, c) when I feel bad about something, my parent will listen, d) if I talk to my parent, I think they try to understand how I feel, e) when I talk to my parent, they make me feel better, and f) if I talk to my parent, they have suggestions about how to handle problems. Responses were averaged and combined (W1: *M* = 3.29, *SD *= 1.33; W2: *M* = 3.17, *SD* = 1.37) and the scale was found to be reliable (W1 Cronbach’s α = .94; W2 Cronbach’s α = .96).

#### 3.4.2. Social media addiction.

Six items were used to measure SMA. These items were acquired from van den Eijnden et al.’s Social Media Disorder Scale [7]. Participants were asked whether during the past year they a) regularly found that they can’t think of anything else but the moment that they will be able to use social media again, b) regularly felt dissatisfied because they wanted to spend more time on social media, c) often felt bad when they could not use social media, d) tried to spend less time on social media, but failed, e) regularly neglected other activities (e.g., hobbies, sports) because they wanted to use social media, and f) often used social media to escape from negative feelings? Consistent with the approach used by van den Eijnden et al. [7], the items were scored with 0 = no and 1 = yes. Responses were averaged and combined (W1: *M* = .43, *SD* = .29; W2: *M* = .47, *SD* = .34) and the scale’s reliability was found to be at an acceptable level (W1 Cronbach’s α = .70; W2 Cronbach’s α = .73).

#### 3.4.3. Depressive symptoms.

The following items, acquired from Revah-Levy et al.’s [48] Adolescent Depression Rating Scale, was used to measure depressive symptoms: a) I have no energy for work/school, b) I have trouble thinking, c) I have felt very sad, d) I have felt like giving up, and e) I sleep badly. We asked participants if they had felt this way in the past week. Responses were measured along 0 = no, 1 = yes. Responses were averaged and combined (W1: *M* = .44, *SD* = .35; W2: *M* = .46, *SD* = .36) and the scale was found to be reliable (W1 Cronbach’s α = .73; W2 Cronbach’s α = .75).

#### 3.4.4. Covariates.

We measured and controlled for age, biological sex, grade, and ethnicity. As mentioned above, because the secondary data came from a pilot study that tested the efficacy of an intervention comprising of two groups, an intervention group and control, we included whether the participant was part of the intervention or control group as a covariate.

### 3.5. Data analysis strategy

We used SPSS version 30 to analyze the descriptive statistics for the demographics and covariates and the mean scores and reliabilities of the key indicators. We used cross-lagged panel design to test the proposed hypotheses [49, 50, 51]. This analysis allows us to estimate the influences of the predictor on the outcome, with the influences of the lagged outcome taken into account. This procedure allows for making more precise predictive inferences than cross-sectional analyses. We took into account age, biological sex, grade levels, condition, and ethnicity (Filipino, Freely Associated States). The errors in the focal variables were allowed to covary within each wave. For all analyses, listwise deletion was used to handle missing data.

To evaluate model fit and test our hypotheses, we used R package, lavaan [52]. We examined the following fit indices – chi-square test, comparative fit index (CFI), standardized root mean square residual (SRMR), and root mean square error of approximation (RMSEA). We used the following criteria as acceptable fit – a value close to.95 for CFI, close to.06 for SRMR, and close to.06-.08 for RMSEA [53,54].

In addition, as part of our investigation for RQ1, we conducted a multivariate analysis of variance (MANOVA) on SPSS Version 30 to examine whether there were differences in the scores for the key variables in both Waves 1 and 2 between the three ethnic groups—CHamoru, Filipino, and FAS. We additionally conducted a multigroup path analysis on R with the three ethnic groups. We first fitted the model for each group to determine whether it was the same across the groups. Next, we constrained the path coefficients to be equal across the groups and tested whether the initial, configural and constrained models differed in model fit. If the constrained model fitted the data better, we freed each hypothesized path coefficient one at a time to determine which path coefficient(s) differed by ethnicity.

## 4. Results

**Fig 1:**
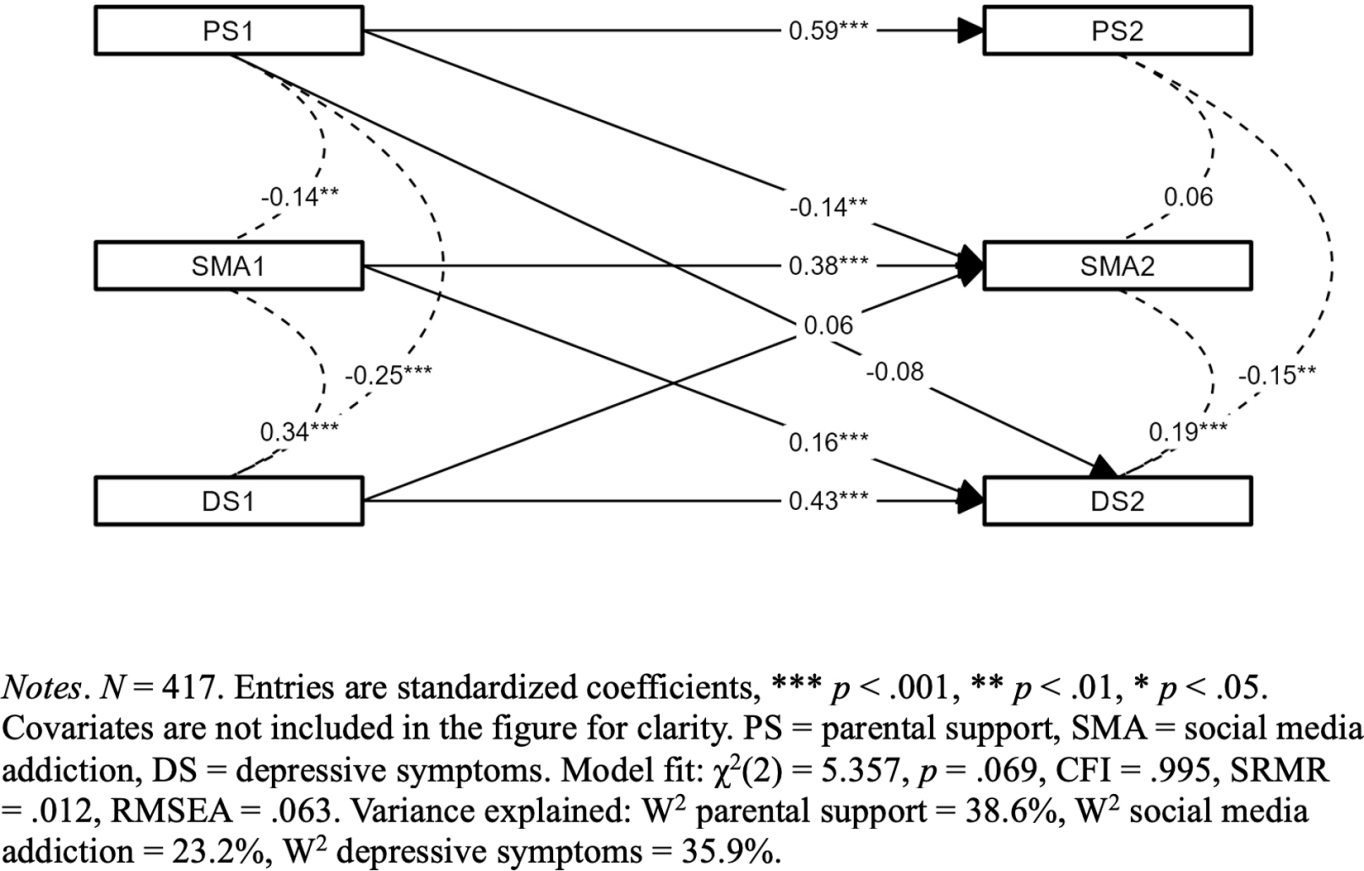
A cross-lagged panel model.

To investigate RQ1, we began by estimating a model with the paths from W1 parental support to W2 social media addiction, from W1 parental support to W2 depressive symptoms, from W1 depressive symptoms to W2 social media addiction, and from W1 social media addiction to W2 depressive symptoms. This model fit the data well: χ^2^(2) = 5.357, *p* = .069, CFI = .995, SRMR = .012, RMSEA = .063. The fit indices met criteria for acceptable model fit, indicating that the model sufficiently reproduced the covariances among these variables. This model is presented in Fig 1. Additionally, although they were not part of our hypotheses, we note that we added the reciprocal paths from W1 social media addiction to W2 parental support and from W1 depressive symptoms to W2 parental support in order to account for the influences of bi-directional relationships. This saturated model did not substantively change the significance and magnitude of the hypothesized paths (Δχ^2^ = 5.357, Δ*df* = 2, *p* = .069).

We summarize the results for RQ1  along four key findings. First, the results showed that W1 PS was not significantly associated with W2 DS (β = -.08, *p* = .063), when W1 DS and W1 SMA were taken into account.

Second, with W1 SMA simultaneously taken into account, W1 PS had a negative association with W2 SMA (β = -.14, *p* = .002). That is, participants who reported a higher level of support from their parents at W1 were less likely to experience SMA at W2.

Third, the path from W1 DS to W2 SMA was not significant (β = .06, *p* = .185). 

Fourth, there was a positive relationship between W1 SMA and W2 DS (β = .16, *p* < .001).

RQ2 explored whether the proposed associations between the key variables (parental support, SMA, and depression) would differ based on ethnicity. First, the results of a MANOVA showed that, between the three ethnic groups (CHamoru, Filipino, and FAS), there were no statistically differences in Wave 1 and Wave 2 scores along the key variables. Next, we performed a multigroup path analysis based on the best fitting model described above. A configural model showed that the model fit the data well for each group: χ^2^(6) = 5.357, *p* = .499 (CHamoru = 0.916, Filipino = 1.742, and FAS = 2.699), CFI = 1.000, SRMR = .013, RMSEA = .000. When all path coefficients were constrained to be equal across the groups, this model fit the data better, χ^2^(68) = 95.552, *p* = .015 (CHamoru = 38.293, Filipino = 27.455, and FAS = 29.804), CFI = .956, SRMR = .051, RMSEA = .055, model fit significantly worsened relative to the unconstrained configural model (Δχ^2^ = 90.195, Δ*df* = 62, *p* = .011), suggesting that the relationship among the focal variables differed across the groups. A further analysis showed, however, that none of the paths significantly differed by ethnicity. Taken together, these findings indicated that although the model was not entirely invariant across the ethnic groups, the specific paths of our interest was not moderated by ethnicity.

## Discussion

Our present study examined the associations between parental support, social media addiction, and depressive symptoms among Guam early adolescents. We particularly analyzed whether PS serves as a protective factor that could mitigate both SMA and depression experienced by Guam adolescents. We analyzed data from two waves of a survey of adolescents in Guam. Our findings extend the literature in a number of areas. First, we found that adolescents who reported a higher level of support from their parents were less likely to experience SMA. This result is consistent with the general benefits model for social support [11] and findings from two recent studies on the association between parental support and problematic internet use [26,27]. In this case, our findings suggest that for Guam adolescents, PS seems to play a protective factor against SMA.

Second, although we expected PS tonegatively associate with DS, our results show that this proposed association was not statistically significant. This lack of a significant association is consistent with a recent longitudinal study that showed that although PS was correlated with DS in the long term, PS did not predict DS in adolescents in the short term [55]. One explanation for this lack of finding is based on the view that PS may be associated with both SMA and DS as a common underlying factor [56]. If such a common underlying mechanism exists, it is possible that PS, SMA, and DS are all correlated, and that the association between PS on DS is washed out after its association with SMA is taken into account. In fact, in our present study, SMA was found to be associated with DS, a finding that we explain directly below. This suggests a potentially stronger association of SMA on DS. In addition, we conducted a cross-lagged panel design in which we accounted for autoregressive associations of DS, which appear to be highly stable over a short-term period. In fact, the correlation between W1 DS and W2 DS was found to be moderate to high. Since our cross-lagged panel model accounted for changes in DS, there may not have been enough room for DS to change over such a short period. On the other hand, SMA may be less stable, allowing us to see some changes due to an independent variable (PS in this study).

Third, our findings showed that SMA was positively associated with DS for Guam adolescents. This finding reinforces previous research showing that addictive uses of the Internet predicts depression [35,39]. Again, our analyses accounted for bi-directionality using cross-lagged panel design. Thus, our results indicated that for Guam adolescents, it is SMA that predicts depressive symptoms, and DS does not predict SMA. Furthermore, our findings do not support the bi-directional association between SMA and depression.

Fourth, and finally, our study indicated that ethnicity—being CHamoru, Filipino, or from the Freely Associated States—did not affect any of the associations between the key variables we examined. This suggests that the statistically significant relationships we found in our study--that PS negatively associates with SMA and SMA positively associates with DS--are consistent regardless of an adolescent’s ethnic background. Thus, irrespective of one’s ethnicity, for youths in Guam, higher levels of PS could be associated with higher SMA. However, regardless of ethnicity, among Guam youths, SMA is positively associated with DS.

Although the present study’s results provide key insights, we acknowledge a number of limitations that could affect the interpretation of our results. First, we emphasize that our cross-sectional design precludes us from making causal inferences or establishing causality. Therefore, we recommend that future research employ experimental designs that are able to establish causality to confirm the direction of the associations we found in our study. Second, our study relied on just two-wave data. However, again, this limitation should be tempered by the fact that we again employed a cross-lagged model, which allowed us to control for autoregressive effects of all variables of interest when estimating the indirect effect. Third, as we alluded to above, the time interval of one month between W1 and W2 may have not been enough to account for changes in our variables of interest. Specifically, PS, SMA, and depression are theoretically dynamic, yet they may have shown limited variability across time in our study, especially given the time interval. This issue may have limited our ability to detect meaningful changes. Thus, we recommend that future studies expand the length between the two Waves (e.g., 6 months or 1 year). Fourth, the covariates we included in our model were not exhaustive, and thus, the possibility of the influence of other variables that we did not account for cannot be ruled out. Past research, for instance, has revealed that variables such as social comparison [37], loneliness [57], and the fear of missing out [58] are correlated with either SMA or DS. Thus, future research might include these variables as covariates in models testing the associations between PS, SMA, and DS. Fifth, we used self-reports to measure our variables, and we should note that self-reporting does not constitute a diagnosis. Finally, as we mentioned in our introduction, SMA is currently not recognized as a formal mental disorder by the DSM-5 or the ICD-11. This thus warrants further research to make a clear distinction between problematic uses of social media with pathological behavior for Guam youths.

With these limitations notwithstanding, our findings provide evidence for the protective role of parental support for social media addiction for Guam youths. Although we expected parental support to also play a supportive role for depressive symptoms for Guam youths, our study revealed that this wasn’t the case when SMA is accounted for. Yet our findings indicated that for Guam youths, the causal direction of the association between SMA and depression appears to be that SMA positively predicts depression, and not vice-versa, nor is the relationship bi-directional. Taken together, these findings could help inform the development of interventions aimed at mitigating both social media addiction and depressive symptoms, particularly, among youths living in communities similar to Guam. That is, when developing interventions for such youths, it is important to address the vital roles played by parental support in potentially decreasing the risk of developing social media addiction, on the one hand, and that of SMA in increasing depression on the other hand. In any case, interventions primarily aimed at preventing or mitigating depression through increasing parental support, could also consider incorporating components aimed at addressing social media addiction.

## Supporting information

S1.Text Results for multivariate analyses of variance to test whether there were statistically significant differences in the mean scores between the intervention and control groups along all our key variables in W1 and W2. (PDF)

S1 Table Caption: Participant characteristics from Wave 1 and Wave 2. (DOCX)
