# Supplementary material for: Associations between parental support, social media addiction, and depressive symptoms among early adolescents in Guam
Source: PLOS Ment Health. 2025 Jun 25;2(6):e0000275. doi: 10.1371/journal.pmen.0000275 (PMC12798394; doi:10.1371/journal.pmen.0000275)
Supplement: S1.Text — (PDF) [file pmen.0000275.s001.pdf]

S1_Text.Results for multivariate analyses of variance to test whether there were statistically significant differences in the mean scores between the intervention and control groups along all key variables in Wave 1 and Wave 2.

General Linear Model

Notes

Output Created		04-APR-2025 18:50:43
Comments		
Input	Data	
	Active Dataset	DataSet1
	Filter	<none>
	Weight	<none>
	Split File	<none>
	N of Rows in Working Data File	710
Missing Value Handling	Definition of Missing	User-defined missing values are treated as missing.
	Cases Used	Statistics are based on all cases with valid data for all variables in the model.
Syntax		GLM t1SocS t1SMDis REVt1_dp t2SocS t2SMDis REVt2_dp BY t1_Con<br>/METHOD=SSTYPE(3)<br>/INTERCEPT=INCLUDE<br>/EMMEANS=TABLES (OVERALL)<br>/EMMEANS=TABLES (t1_Con)<br>/PRINT=DESCRIPTIVE ETASQ<br>/CRITERIA=ALPHA(.05)<br>/DESIGN= t1_Con.
Resources	Processor Time	00:00:00.04
	Elapsed Time	00:00:00.00

Between-Subjects Factors

		N
1 = Intervention, 2 = Control	1	219
	2	208

Descriptive Statistics

	1 = Intervention, 2 = Control	Mean	Std. Deviation	N
Parental support-Wave 1	1	3.3364	1.32799	219
	2	3.2588	1.31555	208
	Total	3.2986	1.32096	427
Social media addiction-Wave 1	1	.4459	.28701	219
	2	.4321	.29537	208
	Total	.4391	.29085	427
Depressive symptoms-Wave 1	1	.4323	.34012	219
	2	.4498	.36619	208
	Total	.4408	.35275	427
Parental support-Wave 2	1	3.2207	1.37349	219
	2	3.0745	1.39916	208
	Total	3.1495	1.38635	427
Social media addiction-Wave 2	1	.4944	.36297	219
	2	.4652	.31963	208
	Total	.4802	.34245	427
Depressive symptoms-Wave 2	1	.4929	.36291	219
	2	.4262	.34015	208
	Total	.4604	.35318	427

Multivariate Tests[a]

Effect		Value	F	Hypothesis df	Error df	Sig.
Intercept	Pillai's Trace	.940	1102.287[b]	6.000	420.000	<.001
	Wilks' Lambda	.060	1102.287[b]	6.000	420.000	<.001
	Hotelling's Trace	15.747	1102.287[b]	6.000	420.000	<.001
	Roy's Largest Root	15.747	1102.287[b]	6.000	420.000	<.001
t1_Con	Pillai's Trace	.024	1.690[b]	6.000	420.000	.122
	Wilks' Lambda	.976	1.690[b]	6.000	420.000	.122
	Hotelling's Trace	.024	1.690[b]	6.000	420.000	.122
	Roy's Largest Root	.024	1.690[b]	6.000	420.000	.122

Multivariate Tests[a]

Effect		Partial Eta Squared
Intercept	Pillai's Trace	.940
	Wilks' Lambda	.940
	Hotelling's Trace	.940
	Roy's Largest Root	.940
t1_Con	Pillai's Trace	.024
	Wilks' Lambda	.024
	Hotelling's Trace	.024
	Roy's Largest Root	.024

a. Design: Intercept + t1_Con

b. Exact statistic

Tests of Between-Subjects Effects

Source	Dependent Variable	Type III Sum of Squares	df	Mean Square	F
Corrected Model	Parental support-Wave 1	.642[a]	1	.642	.367
	Social media addiction-Wave 1	.020[b]	1	.020	.241
	Depressive symptoms-Wave 1	.033[c]	1	.033	.262
	Parental support-Wave 2	2.280[d]	1	2.280	1.187
	Social media addiction-Wave 2	.091[e]	1	.091	.772
	Depressive symptoms-Wave 2	.475[f]	1	.475	3.833
Intercept	Parental support-Wave 1	4640.175	1	4640.175	2655.263
	Social media addiction-Wave 1	82.226	1	82.226	970.252
	Depressive symptoms-Wave 1	82.993	1	82.993	665.820
	Parental support-Wave 2	4227.672	1	4227.672	2200.609
	Social media addiction-Wave 2	98.232	1	98.232	837.180
	Depressive symptoms-Wave 2	90.121	1	90.121	727.321
t1_Con	Parental support-Wave 1	.642	1	.642	.367
	Social media addiction-Wave 1	.020	1	.020	.241

Tests of Between-Subjects Effects

Source	Dependent Variable	Sig.	Partial Eta Squared
Corrected Model	Parental support-Wave 1	.545	.001
	Social media addiction-Wave 1	.624	.001
	Depressive symptoms-Wave 1	.609	.001
	Parental support-Wave 2	.277	.003
	Social media addiction-Wave 2	.380	.002
	Depressive symptoms-Wave 2	.051	.009
Intercept	Parental support-Wave 1	<.001	.862
	Social media addiction-Wave 1	<.001	.695
	Depressive symptoms-Wave 1	<.001	.610
	Parental support-Wave 2	<.001	.838
	Social media addiction-Wave 2	<.001	.663
	Depressive symptoms-Wave 2	<.001	.631
t1_Con	Parental support-Wave 1	.545	.001
	Social media addiction-Wave 1	.624	.001

Tests of Between-Subjects Effects

Source	Dependent Variable	Type III Sum of Squares	df	Mean Square	F
	Depressive symptoms-Wave 1	.033	1	.033	.262
	Parental support-Wave 2	2.280	1	2.280	1.187
	Social media addiction-Wave 2	.091	1	.091	.772
	Depressive symptoms-Wave 2	.475	1	.475	3.833
Error	Parental support-Wave 1	742.704	425	1.748	
	Social media addiction-Wave 1	36.018	425	.085	
	Depressive symptoms-Wave 1	52.976	425	.125	
	Parental support-Wave 2	816.483	425	1.921	
	Social media addiction-Wave 2	49.868	425	.117	
	Depressive symptoms-Wave 2	52.661	425	.124	
Total	Parental support-Wave 1	5389.417	427		
	Social media addiction-Wave 1	118.386	427		
	Depressive symptoms-Wave 1	135.972	427		
	Parental support-Wave 2	5054.306	427		
	Social media addiction-Wave 2	148.410	427		
	Depressive symptoms-Wave 2	143.655	427		
Corrected Total	Parental support-Wave 1	743.346	426		
	Social media addiction-Wave 1	36.038	426		
	Depressive symptoms-Wave 1	53.008	426		
	Parental support-Wave 2	818.763	426		
	Social media addiction-Wave 2	49.959	426		
	Depressive symptoms-Wave 2	53.136	426		

Tests of Between-Subjects Effects

Source	Dependent Variable	Sig.	Partial Eta Squared
	Depressive symptoms-Wave 1	.609	.001
	Parental support-Wave 2	.277	.003
	Social media addiction-Wave 2	.380	.002
	Depressive symptoms-Wave 2	.051	.009
Error	Parental support-Wave 1		
	Social media addiction-Wave 1		
	Depressive symptoms-Wave 1		
	Parental support-Wave 2		
	Social media addiction-Wave 2		
	Depressive symptoms-Wave 2		
Total	Parental support-Wave 1		
	Social media addiction-Wave 1		
	Depressive symptoms-Wave 1		
	Parental support-Wave 2		
	Social media addiction-Wave 2		
	Depressive symptoms-Wave 2		
Corrected Total	Parental support-Wave 1		
	Social media addiction-Wave 1		
	Depressive symptoms-Wave 1		
	Parental support-Wave 2		
	Social media addiction-Wave 2		
	Depressive symptoms-Wave 2		

a. R Squared = .001 (Adjusted R Squared = -.001)

b. R Squared = .001 (Adjusted R Squared = -.002)

c. R Squared = .001 (Adjusted R Squared = -.002)

d. R Squared = .003 (Adjusted R Squared = .000)

e. R Squared = .002 (Adjusted R Squared = -.001)

f. R Squared = .009 (Adjusted R Squared = .007)

Estimated Marginal Means

1. Grand Mean

Dependent Variable	Mean	Std. Error	95% Confidence Interval	
			Lower Bound	Upper Bound
Parental support-Wave 1	3.298	.064	3.172	3.423
Social media addiction-Wave 1	.439	.014	.411	.467
Depressive symptoms-Wave 1	.441	.017	.407	.475
Parental support-Wave 2	3.148	.067	3.016	3.279
Social media addiction-Wave 2	.480	.017	.447	.512
Depressive symptoms-Wave 2	.460	.017	.426	.493

2. 1 = Intervention, 2 = Control

Dependent Variable	1 = Intervention, 2 = Control	Mean	Std. Error	95% ...
				Lower Bound
Parental support-Wave 1	1	3.336	.089	3.161
	2	3.259	.092	3.079
Social media addiction-Wave 1	1	.446	.020	.407
	2	.432	.020	.392
Depressive symptoms-Wave 1	1	.432	.024	.385
	2	.450	.024	.402
Parental support-Wave 2	1	3.221	.094	3.037
	2	3.075	.096	2.886
Social media addiction-Wave 2	1	.494	.023	.449
	2	.465	.024	.419
Depressive symptoms-Wave 2	1	.493	.024	.446
	2	.426	.024	.378

2. 1 = Intervention, 2 = Control

Dependent Variable	1 = Intervention, 2 = Control	95% ... Upper Bound
Parental support-Wave 1	1	3.512
	2	3.439
Social media addiction-Wave 1	1	.485
	2	.472
Depressive symptoms-Wave 1	1	.479
	2	.498
Parental support-Wave 2	1	3.405
	2	3.263
Social media addiction-Wave 2	1	.540
	2	.512
Depressive symptoms-Wave 2	1	.540
	2	.474
