# Supplementary material for: Associations between parental support, social media addiction, and depressive symptoms among early adolescents in Guam
Source: PLOS Ment Health. 2025 Jun 25;2(6):e0000275. doi: 10.1371/journal.pmen.0000275 (PMC12798394; doi:10.1371/journal.pmen.0000275)
Supplement: S1 Table — (DOCX) [file pmen.0000275.s002.docx]

S1 Table. Participant characteristics from Wave 1 and Wave 2.

|  | Wave 1  n = 538 | | Wave 2  n = 507 |
| --- | --- | --- | --- |
|  | Frequency (%) | | |
| Age |  |  | |
| 10 | 1% | 1% | |
| 11 | 17% | 19% | |
| 12 | 28% | 28% | |
| 13 | 38% | 46% | |
| 14 | 16% | 6% | |
|  |  |  | |
| Sex |  |  | |
| Girls | 48% | 53% | |
| Boys | 52% | 47% | |
|  |  |  | |
| Grade |  |  | |
| 6^th^ | 24% | 26% | |
| 7^th^ | 29% | 31% | |
| 8^th^ | 47% | 43% | |
|  |  |  | |
| Ethnicity |  |  | |
| Chamorro | 55% | 48% | |
| Filipino | 19% | 20% | |
| Freely Associated States (FAS) | 17% | 25% | |
| Other | 9% | 7% | |
